# Response of Soil CO_**2**_ Emission and Summer Maize Yield to Plant Density and Straw Mulching in the North China Plain

**DOI:** 10.1155/2014/180219

**Published:** 2014-07-23

**Authors:** Quanru Liu, Xinhui Liu, Chengyue Bian, Changjian Ma, Kun Lang, Huifang Han, Quanqi Li

**Affiliations:** ^1^College of Water Conservancy and Civil Engineering, Shandong Agricultural University, Tai'an, Shandong 271000, China; ^2^State Key Laboratory of Crop Biology, Shandong Key Laboratory of Crop Biology, Shandong Agricultural University, Tai'an, Shandong 271000, China

## Abstract

Demand for food security and the current global warming situation make high and strict demands on the North China Plain for both food production and the inhibition of agricultural carbon emissions. To explore the most effective way to decrease soil CO_2_ emissions and maintain high grain yield, studies were conducted during the 2012 and 2013 summer maize growing seasons to assess the effects of wheat straw mulching on the soil CO_2_ emissions and grain yield of summer maize by adding 0 and 0.6 kg m^−2^ to fields with plant densities of 100 000, 75 000, and 55 000 plants ha^−1^. The study indicated that straw mulching had some positive effects on summer maize grain yield by improving the 1000-kernel weight. Meanwhile, straw mulching effectively controlled the soil respiration rate and cumulative CO_2_ emission flux, particularly in fields planted at a density of 75 000 plants ha^−1^, which achieved maximum grain yield and minimum carbon emission per unit yield. In addition, soil microbial biomass and microbial activity were significantly higher in mulching treatments than in nonmulching treatments. Consequently, summer maize with straw mulching at 75 000 plants ha^−1^ is an environmentally friendly option in the North China Plain.

## 1. Introduction

Climate changes involving warming caused by increased concentrations of CO_2_ in the atmosphere and food security problems owing to fast-growing human populations and loss of farmland have become global issues seriously threatening developing countries. The rate of increase in global mean temperature in response to the change in atmospheric CO_2_ content is known perhaps to a factor of three [1]. Annually, about 75 Pg C is released into the atmosphere through soil respiration. Small changes in the magnitude of soil respiration could have a large effect on the concentration of CO_2_ in the atmosphere [2]. In order to reduce and mitigate the potential negative effects of rising temperatures on ecosystems and human well-being, a series of strategies are needed to reduce CO_2_ emissions and atmospheric CO_2_ concentration [3]. The largest terrestrial organic C sink is the soil and soil C sequestration is a strategy to achieve food security through improvements in soil quality [4]. Cropland ecosystems have a high, yet attainable, soil C sequestration potential.

Soil respiration is complex and variable and is controlled by many abiotic and biotic factors. Various studies have indicated that different factors act as controls on soil respiration at the site level. Soil emissions have previously been shown to increase after application of inorganic fertilizer or incorporation of crop residues [5, 6]. There has been considerable discussion about the responses of soil respiration and C accumulation to N fertilization [7, 8]. Temperature and soil water content have been found to be the dominant factor and limiting factor for soil respiration, respectively [9]; there was a consistently strong correlation between daily mean soil respiration and enhanced vegetation index for maize fields [10]. Application of plant residues to soil has beneficial effects on a number of soil properties, and the return of straw to the soil can sequester 9.76 Tg C each year [11]. Mulch alters the soil microenvironment and has significant effects on many biochemical processes in the soil [12], resulting in an increase of microbial carbon biomass and enzyme activities [13, 14].

The effects of straw mulching on soil carbon emission and sequestration in cropland have not been conclusively agreed upon among reported studies. As noted by Jacinthe et al. (2002), mulch application rate had a significant effect on the seasonal CO_2_ emission of uncropped land with average daily fluxes generally higher in the mulched than in the bare plots [15]. The soil respiration rate of soil surface covered with maize residue was higher than that of nonmulching in fields of grass species and white clover [16]. Annual CO_2_ emissions were higher with corn residues amendment than with no residue [17]. In contrast, cumulative CO_2_ emission was 24% less for no-tillage with residue than without residue in corn-soybean fields [18]. Corn residue mulch with optimum N fertilizer in zero tillage reduced CO_2_ emissions and gave better yields [19]. It is obvious that the effect of straw mulching on soil carbon emissions is not consistent; therefore, further study should be conducted to discuss the effect of mulching on carbon emission and utilization in cropland.

Maize yield varies considerably with different plant densities under different cultivation practices [20]. Seeding rates above the optimum result in lower grain yields because of higher competition between plants [21]. Drought-prone environments require lower plant densities for efficient use of growth resources [22]. Short-duration hybrids should be sowed at higher densities to compensate for the lower leaf area per plant [23]. Grain yield increases as plant density increases. While the rate of increase slows as density increases, the yield is greatest at the highest plant density [24].

Will the application of straw mulching enhance crop productivity and reduce soil CO_2_ emissions at different plant densities? How will soil microbial biomass and microbial activity respond to plant density and straw mulching in the North China Plain? Answering these questions will help to assess the consequences that straw mulching in croplands has on C biological cycling and storage and will be crucial for the development and implementation of sustainable sequestration policies worldwide. Moreover, a previous study on the effects of straw mulching on water consumption characteristics and yield suggested that a 0.6 kg m^−2^ straw mulching quantity [25] be added to one half of the plots. Thus, the objectives of this study were to (I) determine the resulting changes in soil CO_2_ emission and yield; (II) examine how different management regimes affect soil microbial biomass and microbial activity; and (III) build a proper management schedule to enable the soil to serve as a CO_2_ sink.

## 2. Materials and Methods

### 2.1. Experimental Site

The experiment was conducted at the Experimental Station of Shandong Agricultural University (36°10′′9′N, 117°9′′03′E) in the North China Plain in the summer maize growing seasons of 2012 and 2013. The study field is located in a warm, semihumid region with a continental climate. The average annual rainfall is 786.3 mm, and 65.1% of the local rainfall is concentrated in the summer, which can satisfy the water requirement for all growth stages of summer maize. The soil of the experimental site is loamy (40% sand, 44% silt, and 16% clay) with 32.4% field water capacity [26]. In the 2012 and 2013 summer maize growing seasons, total rainfall was 337.1 and 461.8 mm, respectively. The experiment was conducted in plots divided by concrete walls that were 25 cm thick and that extend 1.5 m beneath the surface. The area of the plots was 3 m × 3 m. Visual observations of the experimental plots did not reveal any signs of tillage or water erosion. Therefore, negligible amounts of fertilizer were lost through erosion.

### 2.2. Experimental Design

The summer maize variety used in the experiment was “DengHai 661.” The seeding times of the summer maize were June 17, 2012, and June 19, 2013. Maize was harvested on October 3, 2012, and October 2, 2013. Before sowing, each plot was irrigated with 0.5 m^3^ of water to guarantee all treatments were under the same soil moisture regime. In both summer maize growing seasons, no irrigation was applied. A split plot design randomized complete block was used with two mulching rates in the main plots and three plant density treatments in the subplots (Table 1). Each plot consisted of five rows with 60 cm row spacing, repeated three times. At the summer maize five-leaf stage, straw mulching on soil surface was carried out by applying winter wheat straw that was chopped into 3–5 cm pieces. At the beginning of July, the levels of rapidly available phosphorus, potassium, and nitrogen were applied at a rate of 7.5, 11.3, and 15.0 g m^−2^, respectively, before raining according to forecast.

### 2.3. Measurements

The CO_2_ flux was monitored using a GXH-305 Portable Gas Analyzer (ADC Bioscientific Ltd., Hoddesdon, England) with static chambers for gas sampling made of polyvinyl (PVC) pipe. Gas chamber bases measured 15.7 cm in height and 25 cm in diameter. The chambers were inserted tightly into the ground without removing any of the surface soil when the measurement started. CO_2_ was sampled and analyzed five times during each whole growth period. Each measurement was taken for less than 2 min on a sunny day between 9:00 a.m. and 10:00 a.m. Along with CO_2_ data, air temperature data were also recorded from each treatment 10 cm above the ground surface.

To measure soil microbial biomass and microbial activity, soil samples were collected from five randomly chosen points from each plot at 0–10 cm depths using a hand augur. The samples from each treatment were then mixed together to make a composite sample of each treatment which was then stored at 4°C. We used the GXH-305 Portable Gas Analyzer to determine substrate-induced respiration. To measure soil microbial biomass, soil samples were mixed with glucose (5 *μ*mol g^−1^ soil) and talc powder (0.025 g) incubated for 2 h at 22°C in three replicates [27]. Soil microbial biomass was calculated at the maximum initial respiration response using the following equation:

(1) $$\begin{array}{c} x=40.04y+0.37, \end{array}$$

where *x* is the microbial biomass (*μ*g CO_2_-C g^−1^ soil h^−1^) and *y* is the amount of soil respiration (*μ*L CO_2_ g^−1^ soil h^−1^).

The soil microbial activity was the amount of soil respiration which was measured by the GXH-305 Portable Gas Analyzer without the addition of substrate for 24 h at 22°C in three replicates and was expressed as *μ*g CO_2_-C g^−1^ soil h^−1^.

Maize grain yield was determined by harvesting the two central lines of each plot. Spike numbers per m^2^, rows per spike, kernels per row, and 1000-kernel weight at 12% moisture content were also recorded.

Soil respiration rate was measured by soil surface CO_2_ flux which was calculated as follows:

(2) $$\begin{array}{c} F=\rho \times \frac{V}{A}\times 100\times \frac{P}{P_{0}}\times \frac{273}{273+T}\times \frac{dC}{dt}\times 60, \end{array}$$

where *F* is the soil surface CO_2_ flux (*μ*g m^−2^ h^−1^); *ρ* is the density of CO_2_ under standard atmospheric condition (mg m^−3^); *V* is the volume of static chambers (cm^3^); *A* is the area of static chambers (cm^2^); *P* is the atmospheric pressure in static chambers (Pa); *P*_0_ is the atmospheric pressure under standard atmospheric condition (1.013 × 10^5^ Pa) and the atmospheric pressure in Tai'an is roughly equal to that under standard atmospheric condition; *T* is the atmospheric temperature (°C); *dC*/*dt* is change in concentration of CO_2_ (10^−6^ min^−1^).

Cumulative emissions of CO_2_-C were calculated as follows [28]:

(3) $$\begin{array}{c} M=\frac{\sum \left(F_{i+1}+F_{i}\right)}{2}\times \left(t_{i+1}-t_{i}\right)\times 24, \end{array}$$

where *M* is cumulative emissions of CO_2_-C (mg C m^−2^); *F* is soil surface CO_2_ flux (kg CO_2_ ha^−1^ h^−1^); *i* is the sampling number; *t* is the day after sowing.

The amount of CO_2_ emission per unit grain yield was calculated as follows [29]:

(4) $$\begin{array}{c} R=\frac{M}{Y}, \end{array}$$

where *M* is the cumulative emissions of CO_2_-C (kg km^−2^); *Y* is the grain yield (kg km^−2^).

### 2.4. Statistical Analysis

An analysis of variance was completed on the treatments. Multiple comparisons of annual mean values were performed by the least-significant difference method (LSD). In all analyses, a probability of error smaller than 5% (*P* < 0.05) was considered significant. The Origin 8.0 software was used for all drawings [30].

## 3. Results

### 3.1. Soil Respiration Rate

**Figure 1:**
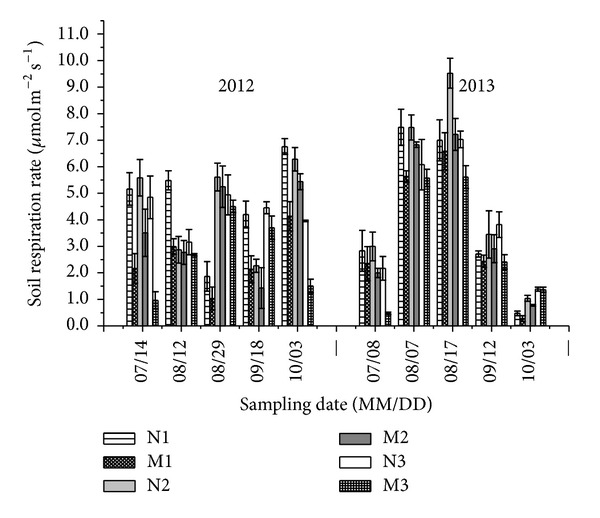
Soil respiration rate of different treatments in 2012 and 2013 summer maize growing seasons. Vertical bars represent standard error.

In these experiments, soil respiration rates of the different treatments ranged between 0.97 and 6.75 *μ*mol m^−2^ s^−1^ in 2012 and 0.48 and 9.52 *μ*mol m^−2^ s^−1^ in 2013 (Figure 1). In both growing seasons, regardless of whether the mulching treatment was applied, the variation trend in soil respiration rate in the different plant densities was not consistent. However, within the same plant density, the soil respiration rate in the presence of the mulching treatment was much higher than that in nonmulching treatments. The greatest difference between the mean soil respiration rate of the mulching treatments (i.e., M1, M2, and M3) and nonmulching treatments (i.e., N1, N2, and N3) was on July 14, 2012, and July 8, 2013. The mean soil respiration rates in soils subjected to mulching treatments were 35.37% and 19.89% lower than those of nonmulching treatments in 2012 and in 2013 growing seasons, respectively.

### 3.2. Cumulative CO_2_ Emissions Flux

**Figure 2:**
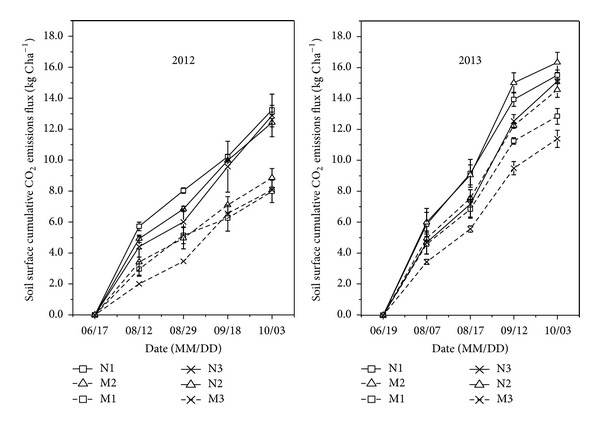
The dynamic change of cumulative CO_2_ emissions flux in 2012 and 2013 summer maize growing seasons. Vertical bars represent standard error.

Figure 2 illustrates the dynamic changes in cumulative emissions of CO_2_-C in the 2012 and 2013 summer maize growing seasons. It shows that the soil surface CO_2_ fluxes of nonmulching treatments were significantly higher than those of mulching treatments for the same plant density. The highest soil surface CO_2_ flux recordings were observed in N1 (13.24 kg CO_2_ ha^−1^) in 2012 and in N2 (16.34 kg CO_2_ ha^−1^) in 2013, and the lowest were both recorded in M3 (8.08 and 11.40 kg CO_2_ ha^−1^, in 2012 and in 2013, resp.).

### 3.3. Soil Microbial Biomass and Microbial Activity

**Figure 3:**
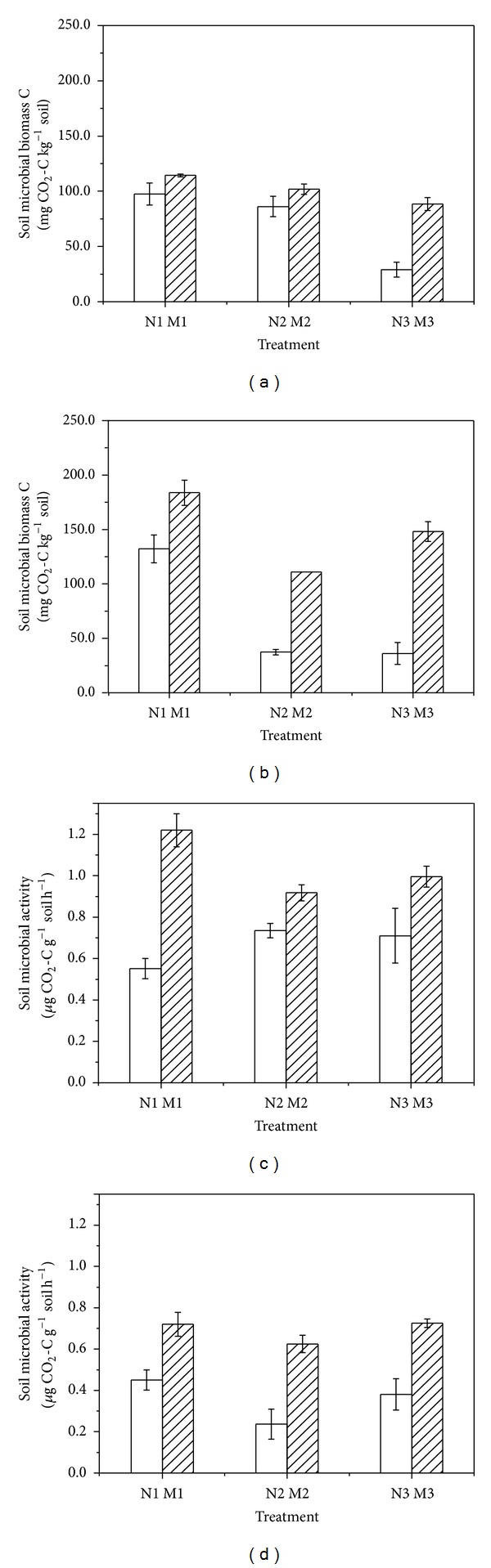
The mean microbial biomass and activity of different treatments at filling stage, 2013 ((a), (c)), and maturity stage, 2013 ((b), (d)). Vertical bars are standard errors.

Microbial biomass and microbial activity of soils with mulching treatments were significantly higher than those of nonmulching treatments at both filling and maturity stages (Figure 3). The highest microbial biomass was observed in M1 (114.33 mg CO_2_-C kg^−1^ soil) at the maturity stage and the lowest in N3 (28.86 mg CO_2_-C kg^−1^ soil) at the filling stage; and the highest microbial biomass was observed in M1 (1.22 *μ*g CO_2_-C g^−1^ soil h^−1^) at the filling stage and the lowest in N2 (0.24 *μ*g CO_2_-C g^−1^ soil h^−1^) at the maturity stage. Overall, the soil microbial biomass showed an increasing trend from filling to maturity stages, while the soil microbial activity showed the opposite trend.

### 3.4. Grain Yield and Yield Components

Summer maize grain yield and yield components are presented in Table 2. The highest grain yield in each of the two growing seasons was observed in M2 (1255.44 and 1168.85 g m^−2^, in 2012 and in 2013, resp.) and the lowest in M3 (936.13 and 878.06 g m^−2^, in 2012 and in 2013, resp.). In low plant density, no differences were observed in all yield components between M1 and N1 for either growing season, which resulted in no significant difference in grain yield. However, both in medium and in high plant densities, the grain yields in the mulching treatments were significantly higher than those in the nonmulching treatments between plants of the same plant density. This result was mainly due to the fact that 1000-kernel weight was significantly different between the mulching and nonmulching treatments. Regardless of whether the plants were in low, medium, or high plant densities, there were no differences in spike numbers, rows per spike, and kernels per row between the mulching and nonmulching treatments. Hence, the results show that straw mulching can significantly improve the grain yield both in medium and in low plant densities by improving the 1000-kernel weight.

Compared with nonmulching treatments, the grain yield of the mulching treatments was significantly higher in both growing seasons, which was mainly due to the difference in 1000-kernel weight. There was, however, no statistically significant difference in rows per spike or kernels per row. There were significant differences in kernel numbers and kernels per row between the three density treatments. However, the significant difference in kernels per row between the high- and medium-density was offset by the difference in kernel numbers in these plants. As a result, no significant difference in grain yield was observed between the high- and medium-density treatments in either the 2012 or the 2013 summer maize growing seasons.

### 3.5. Amount of CO_2_ Emission per Unit Grain Yield

**Figure 4:**
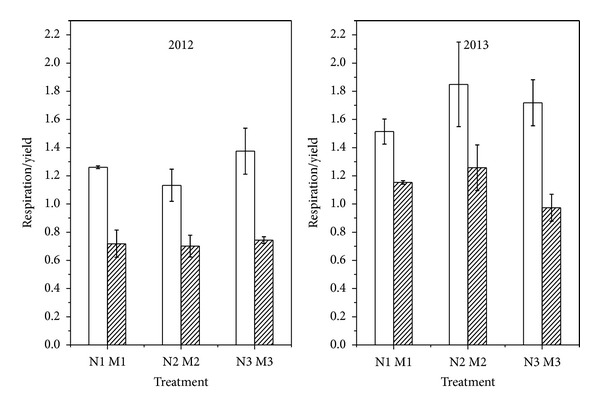
The respiration/yield ratio of different treatments in 2012 and 2013 summer maize growing seasons. Vertical bars are standard errors.

Figure 4 shows the amount of CO_2_ released into the atmosphere per unit grain yield. The result showed that the amount of CO_2_ emission per unit grain yield was significantly lower in mulching treatments than in nonmulching treatments. M1, M2, and M3 were lower by 43.01%, 38.07%, and 45.90% in 2012 and 23.83%, 31.99%, and 43.40% in 2013 than those in N1, N2, and N3, in the respective years. Furthermore, the amount of CO_2_ emission per unit grain yield in all treatments in 2013 was higher than that in 2012. The increases in the treatments were by 20.10%, 60.64%, 63.27%, 79.31%, 24.92%, and 30.70%, in N1, M1, N2, M2, N3, and M3, respectively. It showed no significant difference between the various plant density treatments in the same mulching treatment.

## 4. Discussion

Soil respiration reflects the relationship between C emissions and crop growth and development. Soil mulching has a beneficial effect on soil organic content sequestration and strongly influences the temporal pattern of CO_2_ emission from soils [15]. Results from this study demonstrated that straw mulching could affect soil respiration rate, CO_2_ emission flux, and grain yield in summer maize growing seasons. These findings corroborate previous results that showed that treatment involving no-tillage with surface residue could further reduce CO_2_ emission compared to no-tillage without residue [19]. Both the conversion of residue-C to soil organic carbon and the mineralization of soil organic carbon are accompanied by the generation of CO_2_, contributing to extra return of CO_2_ to the atmosphere. Higher root respiration and microbial respiration using native soil organic carbon and exudates as substrates may also cause more soil CO_2_ emission in the absence of N fertilization [31]. Greater allocation of carbohydrates to improve root growth and maintain root respiration could reduce crop growth and final grain yield [32]. Since our experiment was conducted at low nitrogen levels, the reduction of soil respiration rate and cumulative CO_2_ emissions flux in straw mulching treatments in this study may also be related to decreases in root respiration and the absence of N fertilization.

About 50–70% of the soil respiration of CO_2_ is derived from soil microbes [2]. In this study, straw mulching treatments generally increased soil microbial biomass C and activity as compared to nonmulching treatments. This result is the same as that obtained in organic tomato farming systems [33], where straw mulching further enhanced microbial respiration compared to nonmulched soils. Surface mulching of residues is conducive to denitrification under the residues by increasing the soil water content, supplying available C indicated by high measured microbial activity, and most likely creating anaerobic micro sites [34]. Other reported data show that soil temperature and water content influenced microbial activities, microbial respiration, and C mineralization, thereby resulting in different CO_2_ emission fluxes [35, 36]. The relationship between soil temperature and water content and soil respiration components need an intensive study.

This study clearly showed that mulching treatments had achieved an overall greater grain yield as compared to nonmulching treatments by increasing the 1000-kernel weight. The difference between mulched soil and bare soil with maize plants is not due to the total water consumption but is due to the alteration of the ratio of transpiration to evapotranspiration [37]. The mechanisms of transpiration and evapotranspiration, in coordination with carbon utilization, and their respective impacts on grain yield of different mulching treatments and plant densities should be studied further. Many studies have found that the higher grain yield was directly related to the higher number of spikes per unit area [38, 39], and higher seeding rate elevated grain yield even though increased density reduced kernel weight [40]. However, our study shows that increases in the number of kernels per row offset the decrease in the number of spikes per unit area leading to no difference in grain yield between high- and medium-density treatments. Kernels per row decreased significantly with increasing seeding density.

With respect to the amount of CO_2_ emission per unit grain yield, straw mulching treatments were significantly lower than nonmulching treatments but showed no significant difference in the different plant densities. The same was true for grain yield. Elevated CO_2_ appears not to enhance the yield of maize, accounting for moderate variations in soil fertility and drought stress [41]. Thus, even though mulching treatments reduced CO_2_ emission to the atmosphere compared to nonmulching treatments, the production was higher due to ample supply of water. The amount of CO_2_ emission per unit grain yieldin 2013 showed an upward trend, and this may be caused by an increase in temperature, which is considered a secondary impact of potential climate change due to elevated CO_2_ [42]. Taking the sequestered carbon into account, the lower emission amount per unit yield is likely owing to the reduction in soil respiration caused by root respiration restraint and the increase in the crops' use of soil carbon can be attributed to higher soil microbial biomass and activity. To address this issue, several additional explorations are still needed.

## 5. Conclusion

The 2-year experiment's results showed that straw mulching had some positive effects on summer maize grain yield by improving its 1000-kernel weight. The significant difference in kernels per row between the high- and medium-density was offset by the difference in kernel numbers in these plants. Mulching could reduce the soil respiration rate, limit soil carbon emission, and improve soil microbial biomass carbon and microbial activity in the soil layer 0–10 cm below the surface. The soil microbial biomass shows an increasing trend from filling to maturity stages, which was contrary to the trend of soil microbial activity. In addition, mulching treatments and growth at a density of 75 000 plants ha^−1^ achieved maximum grain yield and minimum carbon emission per unit yield in the North China Plain. Therefore, we suggest that summer maize should be grown with mulching at a density of 75 000 plants ha^−1^ in this region.

## Figures and Tables

**Table 1:** Treatments with the straw mulching and plant density for summer maize.

Straw mulching (kg m^−2^)	Plant density (plants ha^−1^)	Code	Planting distance (cm)
0.6	100 000	M1	17.1
	High-density		
	75 000	M2	22.2
	Medium-density		
	55 000	M3	31.7
	Low-density		

0	100 000	N1	17.1
	High-density		
	75 000	N2	22.2
	Medium-density		
	55 000	N3	31.7
	Low-density		

**Table 2:** Grain yield and yield components under different mulching and density treatments in 2012 and 2013 summer maize growing seasons.

Treatment	Spike number		Rows per spike		Kernels per row		1000-kernel weight		Grain yield	
	(spikes m^−2^)		(rows spike^−1^)		(Kernels row^−1^)		(g)		(g m^−2^)	
	2012	2013	2012	2013	2012	2013	2012	2013	2012	2013
M1	9.67^a^	9.89^a^	16.78^a^	15.39^ab^	27.37^d^	27.45^c^	294.18^c^	309.04^b^	1143.92^ab^	1140.01^ab^
N1	9.63^a^	9.81^a^	16.11^ab^	14.44^b^	27.81^cd^	26.92^c^	296.71^c^	296.13^bc^	1083.53^b^	969.73^bc^
M2	7.93^b^	7.63^b^	16.29^ab^	16.17^a^	30.85^b^	30.16^b^	326.03^b^	325.7^ab^	1255.44^a^	1168.85^a^
N2	7.63^c^	7.63^b^	15.82^b^	16.06^a^	29.52^bc^	29.28^bc^	299.82^c^	265.3^c^	1043.53^bc^	906.25^c^
M3	5.44^d^	5.63^c^	16.63^ab^	16.33^a^	35.37^a^	35.7^a^	343.11^a^	358.92^a^	1072.95^b^	1159.73^a^
N3	5.41^d^	5.22^d^	16.67^a^	16.28^a^	33.85^a^	35.33^a^	322.08^b^	315.82^b^	936.13^c^	878.06^c^

M	7.67^a^	7.72^a^	16.56^a^	15.96^a^	31.20^a^	31.10^a^	321.10^a^	331.22^a^	1157.44^a^	1156.19^a^
N	7.57^a^	7.56^b^	16.20^a^	15.59^a^	30.39^a^	30.51^a^	306.20^b^	292.42^b^	1021.06^b^	918.01^b^
1	9.65^a^	9.85^a^	16.44^ab^	14.92^b^	27.59^c^	27.19^c^	295.45^c^	302.59^b^	1113.72^a^	1054.87^a^
2	7.78^b^	7.63^b^	16.06^b^	16.11^a^	30.19^b^	29.72^b^	312.92^b^	295.50^b^	1149.49^a^	1037.55^a^
3	5.43^c^	5.43^c^	16.65^a^	16.31^a^	34.61^a^	35.52^a^	332.59^a^	337.37^a^	1004.54^b^	1018.89^a^

Means in the same column followed by different letters denote significant differences (LSD, *P* < 0.05).
